# A non-synonymous variant *rs12614* of complement factor B associated with risk of chronic hepatitis B in a Korean population

**DOI:** 10.1186/s12881-020-01177-w

**Published:** 2020-12-17

**Authors:** Jung Yeon Seo, Joong-Gon Shin, Byeong Ju Youn, Suhg Namgoong, Hyun Sub Cheong, Lyoung Hyo Kim, Ji On Kim, Hyoung Doo Shin, Yoon Jun Kim

**Affiliations:** 1 Current address: Department of Core Technology, R&D Center, LG Household & Healthcare (LG H&H), Seoul, 07795 South Korea; 2 Department of Life Science, Sogang University, Seoul, 04107 Republic of Korea; 3 Research Institute for Basic Science, Sogang University, Seoul, 04107 Republic of Korea; 4 Department of Genetic Epidemiology, SNP Genetics Inc., Seoul, 04107 Republic of Korea; 5 Department of Internal Medicine and Liver Research Institute, Seoul National University, 103 Daehak-ro, Jongno-gu, Seoul, 03080 Republic of Korea

**Keywords:** *CFB*, Genetic risk scores, Hepatitis B, Korean population, Liver disease

## Abstract

**Background:**

Hepatitis B is known to cause several forms of liver diseases including chronic hepatitis B (CHB), and hepatocellular carcinoma. Previous genome-wide association study of CHB risk has demonstrated that *rs12614* of *complement factor B* (*CFB)* was significantly associated with CHB risk. In this study, fine-mapping study of previously reported GWAS single nucleotide polymorphism (SNP; *CFB rs12614*) was performed to validate genetic effect of *rs12614* on CHB susceptibility and identify possible additional causal variants around *rs12614* in a Korean population. This association study was conducted in order to identify genetic effects of *CFB* single nucleotide polymorphisms (SNPs) and to identify additional independent CHB susceptible causal markers within a Korean population.

**Methods:**

A total of 10 *CFB* genetic polymorphisms were selected and genotyped in 1716 study subjects comprised of 955 CHB patients and 761 population controls.

**Results:**

A non-synonymous variant, *rs12614* (Arg32Trp) in exon2 of *CFB,* had significant associations with risk of CHB (odds ratio = 0.43, *P* = 5.91 × 10^− 10^). Additional linkage disequilibrium and conditional analysis confirmed that *rs12614* had independent genetic effect on CHB susceptibility with previously identified CHB markers. The genetic risk scores (GRSs) were calculated and the CHB patients had higher GRSs than the population controls. Moreover, OR was found to increase significantly with cumulative GRS.

**Conclusions:**

*rs12614* showed significant genetic effect on CHB risk within the Korean population. As such *rs12614* may be used as a possible causal genetic variant for CHB susceptibility.

**Supplementary Information:**

The online version contains supplementary material available at 10.1186/s12881-020-01177-w.

## Background

Hepatitis B, caused by the hepatitis B virus (HBV) infection, is known to cause several liver diseases including chronic hepatitis B (CHB), cirrhosis, and hepatocellular carcinoma (HCC) [1, 2]. According to the 2015 WHO report, HBV infection affects 3.5% of the world population (257 million individuals) and is especially prevalent in Asian populations [3]. Several genome-wide association studies (GWASs) on CHB risk have been conducted on Asian populations and have found that CHB risk associated loci are typically located in human leukocyte antigen (HLA) regions, such as *HLA-DP* and *HLA-DQ* [4–7], in Chinese, Japanese, and Korean populations. According to our previous GWASs, several genes in HLA regions, such as *transcription factor 19* (*TCF19*), and *valyl-tRNA synthetase 2* (*VARS2*)-*surfactant associated 2* (*SFTA2*), *euchromatin histone-lysine-methyltransferase 2* (*EHMT2*), showed significant genetic effects on CHB susceptibility in a Korean population [5–9]. Interestingly, genetic variants of *CFB*, found on the nearby gene of *EHMT2,* have strong association with CHB susceptibility in Chinese studies [10–12].

*CFB* located on HLA genomic region is essential for regulating T-cell mediated innate immunity in the complement system [13–15]. A number of studies have demonstrated that *CFB* genetic variants are also associated with several diseases related to innate immune responses, anterior uveitis and Vogt-Koyanagi-Harada disease [16, 17]. This study conducted association analysis between *CFB* SNPs and CHB susceptibility to validate genetic effect of *rs12614* and identify possible additional causal variants around *rs12614* in a Korean population by fine-mapping of *CFB* region. Furthermore, the genetic risk scores (GRSs) of all known CHB risk makers were calculated to investigate the cumulative genetic effects of CHB susceptibility in individuals.

## Methods

### Study subjects

In this study, a total of 1716 subjects which were consist of 955 cases and 761 controls were recruited and investigated for identifying genetic effects on CHB. The 955 patients with CHB were obtained from the outpatient clinic of the Liver Unit and the Center for Health Promotion at Seoul National University Hospital, Ajou University Medical Center (Suwon, Korea), and Ulsan University Hospital (Ulsan, Korea). Among the CHB patients, 296 patients were also diagnosed with HCC. The 761 population controls (PCs) were provided by Korea BioBank, the Center for Genome Science, Korea Centers for Disease Control and Prevention, and the National Institute of Health. Seropositivity of the hepatitis B surface (HBsAg; Enzygnost® HBsAg 5.0; Dade Behring, Marburg, Germany) over a 6-month period was used for inclusion criterion for diagnosing patients with chronic HBV infection (Supplementary Table 1). The detailed experimental procedures for HBsAg detection using Enzygnost® HBsAg 5.0 Kit assay is described in elsewhere [18]. Diagnosis of HCC was based on imaging findings of nodules that were larger than 1 cm, showing intense arterial uptake, followed by washout of contrast in the venous-delayed phases, in a 4-phase multi-detector CT scan or dynamic contrast enhanced MRI and/or biopsy [19]. Though individuals with HBsAg (−) and anti-HBc (+) (spontaneously cleared for viral infection) are the best disease controls, individuals with an unknown response to HBV infection were used as the population controls, and some of them still have a chance to CHB and/or HCC when exposed to HBV. The study protocol complied with the Declaration of Helsinki. The study was approved by the institutional review board of Seoul National University Hospital, Ajou University Medical Center, and Ulsan University Hospital. All the subjects participating in the study provided written informed consent.

### SNP genotyping

Following criteria were adopted for SNP selection: 1) Candidate SNPs of the genomic region around *CFB* (CFB gene with 2 kb upstream (to include promoter region) and 500 bp downstream regions; Chr6: 31,911,721-31,920,361) were extracted from 1000 genomes Japanese and Han Chinese data, and minor allele frequency (MAF) and linkage disequilibrium (LD) status of the extracted SNPs were calculated. 2) Using NCBI dbSNP, investigate the functional location of the SNPs (upstream variant (2 kb), 5-prime UTR variant, missense, synonymous-codon, intron variant, downstream variant (500 bp), 3-prime UTR variant) extracted in 1). 3) Based on the 1) and 2), among the SNPs with high LD (*r*^*2*^ > 0.98), SNP with relatively frequent (MAF > 5%) and functional effect based on the position was selected. And promoter region and non-synonymous SNPs with low frequency (MAF ≤ 5%) are additionally selected. As results, 5 tagging SNPs (*rs1048709, rs537160, rs541862, rs4151657* and *rs2072633*) were selected along with 5 non-synonymous SNPs (*rs4151667, rs12614, rs641153, rs117314762* and *rs45484591*). A total of 10 SNPs were genotyped in 955 CHB patients and 761 healthy controls. Genotyping reactions were performed by using BioMark HD system (Fluidigm 192.24 SNPtype™, San Francisco, CA, USA). The primer pools were designed for Specific target amplifications, Allele-specific and Locus-specific primers to detect candidate SNPs, and all the primers for 10 investigated SNPs in this study were designed and provided by the manufacturer of Fluidigm system (Fluidigm Corp., San Francisco, CA, USA). The additional workflow was followed by the manufacturer’s instructions for using the Integrated IFC Controller RX, FC1 Cycler, and EP1 Reader. Signal intensities for genotyping calling were scanned using the EP1 data collection and SNP Genotyping analysis software (Fluidigm Corp., San Francisco, CA, USA). The locations of the investigated SNPs are shown in Supplementary Figure 1A.

### Statistical analysis

LD status of the investigated SNPs were calculated with examination of Lewontin’s D’ (|D’|) and the LD coefficient r2 between all pairs of bi-allelic loci using Haploview v4.2 (http://www.broadinstitute.org/mpg/haploview) [20]. Comparison of genotype distributions, including MAF and Hardy-Weinberg Equilibrium (HWE), between CHB patients and controls and and calculating odds ratios (ORs), 95% confidence intervals, and corresponding *P*-values was carried out with a logistic regression model adjusted for age (continuous value) and sex (male = 0, female = 1) as covariates using SAS, version 9.4 (SAS Inc., Cary, NC, USA). In corrections for multiple comparisons, Bonferroni correction for multiple testing was applied. Conditional logistic regression analysis was performed to investigate whether the novel significant association signal of investigated SNP was independent or affected by previously known CHB markers. Allele test based on the allele distribution of each SNP was also performed to assess the detailed genetic effects. Ten previously reported CHB susceptible loci in a Korean population (*rs9277535* of *HLA-DPB1*; *rs3077* of *HLA-DPA1*; *rs2856718* of *HLA-DQB1*; *rs7453920* of *HLA-DQB2*; *rs1419881* of *TCF19*; *rs1265163* of *OCT4*; *rs652888* and *rs35875104* of *EHMT2*; *rs9394021* and *rs2517459* of *VARS2-SFTA*) [5–9] were used for the conditional analysis and allele test. Based on the results from allele test, GRSs were calculated. The detailed calculation method for GRSs was described in elsewhere [18].

## Results

### Genotyping of *CFB* genetic variants

A total of 10 *CFB* SNPs were selected and genotyped in 1716 Korean subjects, comprised of 955 CHB patients and 761 population controls (Supplementary Table 1). Patients were divided into two subgroups, 659 HCC (−) CHB cases and 296 HCC (+) CHB cases. A gene map and LD among investigated SNPs are shown in Supplementary Figure 1A and B. Detailed information on the investigated SNPs, such as chromosome, position, allele, genotype distribution, heterozygosity, and HWE *P*, are presented in Supplementary Table 2.

### Association of *CFB* genetic polymorphisms with CHB risk

In order to investigate the association between *CFB* genetic polymorphisms and risk of CHB, logistic regression analysis under an additive model was conducted. Analysis results indicated that *rs12614* was significantly associated with risk of CHB even after applying Bonferroni correction for multiple testing (OR = 0.43, *P* = 5.91 × 10^− 10^, *P*_*corr*_ = 2.36 × 10^− 8^; Table 1). In order to validate the genetic effects of *rs12614*, association analysis was conducted using the training and test sets from the subjects in this study (Supplementary Table 3). Additional subgroup analysis was performed to investigate the association between *CFB* SNPs and CHB-related HCC progression. Again, analysis results found that, *rs12614* had significant associations with risk of CHB in both the HCC (−) CHB and the HCC (+) CHB subgroups (*P* = 6.60 × 10^− 8^ and 3.10 × 10^− 6^, respectively) even after Bonferroni correction was applied for multiple testing (*P*_*corr*_ = 2.64 × 10^− 6^ and 1.24 × 10^− 4^, respectively). However, *rs12614* did not show significant genetic effect on CHB-related HCC progression (*P* > 0.05).

**Table 1:** Association of *CFB* genetic polymorphisms with the risk of CHB and HCC

Marker	MAF					Comparing groups										
	Total (*n* = 1716)	CHB			PC (*n* = 761)	CHB vs. PC			HCC (−) vs. PC			HCC (+) vs. PC			HCC (−) vs. HCC (+)	
		All CHB (*n* = 955)	HCC (−) (*n* = 659)	HCC (+) (*n* = f)												
						OR (95% CI)	P*	*P*_corr_^**^	OR (95% CI)	P*	*P*_corr_^**^	OR (95% CI)	P*	*P*_corr_^**^	OR (95% CI)	P*
*rs4151667*	0.018	0.019	0.018	0.022	0.016	1.23 (0.73–2.08)	0.42	–	1.16 (0.65–2.06)	0.61	–	1.41 (0.70–2.80)	0.34	–	0.82 (0.41–1.63)	0.58
*rs12614*	0.078	0.053	0.054	0.048	0.112	0.43 (0.33–0.57)	**5.91 × 10**^**−10**^	**2.36 × 10**^**−8**^	0.45 (0.33–0.61)	**6.60 × 10**^**− 8**^	**2.64 × 10**^**−6**^	0.39 (0.25–0.60)	**3.10 × 10**^**− 6**^	**1.24 × 10**^**−4**^	1.13 (0.72–1.78)	0.58
*rs641153*	0.082	0.083	0.086	0.076	0.080	1.04 (0.81–1.33)	0.74	–	1.08 (0.83–1.41)	0.54	–	0.94 (0.66–1.34)	0.75	–	1.14 (0.80–1.62)	0.45
*rs117314762*	0.015	0.013	0.014	0.010	0.018	0.67 (0.38–1.17)	0.16	–	0.73 (0.40–1.34)	0.31	–	0.54 (0.22–1.32)	0.15	–	1.35 (0.53–3.45)	0.51
*rs1048709*	0.279	0.269	0.265	0.277	0.293	0.88 (0.76–1.02)	0.11	–	0.86 (0.73–1.02)	0.09	–	0.92 (0.74–1.14)	0.47	–	0.94 (0.75–1.16)	0.58
*rs537160*	0.333	0.327	0.322	0.340	0.341	0.93 (0.81–1.08)	0.39	–	0.91 (0.77–1.07)	0.26	–	0.99 (0.81–1.21)	0.95	–	0.91 (0.74–1.13)	0.43
*rs541862*	0.082	0.083	0.086	0.076	0.080	1.04 (0.81–1.33)	0.74	–	1.08 (0.83–1.41)	0.54	–	0.94 (0.66–1.34)	0.75	–	1.14 (0.80–1.62)	0.45
*rs4151657*	0.323	0.335	0.341	0.321	0.307	1.13 (0.98–1.30)	0.09	–	1.16 (0.99–1.36)	0.06	–	1.06 (0.86–1.30)	0.55	–	1.09 (0.88–1.34)	0.40
*rs45484591*	0.001	0.001	0.002	0.000	0.001	0.79 (0.11–5.66)	0.82	–	1.15 (0.16–8.22)	0.89	–	–	–	–	–	–
*rs2072633*	0.477	0.468	0.467	0.471	0.488	0.92 (0.80–1.05)	0.25	–	0.91 (0.79–1.06)	0.26	–	0.93 (0.77–1.13)	0.50	–	0.98 (0.80–1.19)	0.85

Significant associations are shown in bold face (*P* < 0.05)

*MAF* minor allele frequency, *CHB* chronic hepatitis B, *HCC* hepatocellular carcinoma, *OR* odds ratio, *CI* confidence interval

^*^ *P*-value of logistic regression analysis under additive model by adjusting for sex and age as covariates

**P-value after Bonferroni correction for multiple testing

### Independent genetic effect of *rs12614* on CHB risk

In order to understand the association between *rs12614* and CHB risk, particularly with respect to independent genetic effect on CHB susceptibility, this study conducted LD calculations and conditional analysis on 10 previously identified CHB susceptibility markers (*rs9277535* of *HLA-DPB1*; *rs3077* of *HLA-DPA1*; *rs2856718* of *HLA-DQB1*; *rs7453920* of *HLA-DQB2*; *rs1419881* of *TCF19*; *rs1265163* of *OCT4*; *rs652888* and *rs35875104* of *EHMT2*; *rs9394021* and *rs2517459* of *VARS2-SFTA*). Supplementary Figure 2 shows the LD plot of *rs12614* and the 10 CHB susceptibility markers. The results show that *CFB rs12614* did not display tight LDs with any known, nearby CHB-susceptible loci (pairwise *r*^*2*^ ≤ 0.15; Supplementary Figure 2). In addition, when adjusting for previously identified CHB markers, *rs12614* maintained significant association with CHB risk (*P* < 0.05; Table 2), indicating that *rs12614* had independent genetic effect on CHB susceptibility to previously identified CHB risk markers.

**Table 2:** Results of conditional analysis with the previously identified CHB marker

Marker	Allele	OR (95% CI)	*P*-value*	Conditional *P*-value by									
				*HLA-DPB1* rs9277535**	*HLA-DPA1* rs3077**	*HLA-DQB1* rs2856718**	*HLA-DQB2* rs7453920**	*TCF19* rs1419881**	*OCT4* rs1265163**	*EHMT2* rs652888**	*EHMT2* rs35875104**	*VARS2-SFTA* rs9394021**	*VARS2-SFTA* rs2517459**
*rs12614*	C > T	0.42 (0.31–0.56)	**5.91 × 10**^**−10**^	**5.94 × 10**^**−6**^	**3.52 × 10**^**−5**^	**4.46 × 10**^**− 6**^	**1.57 × 10**^**−4**^	**4.73 × 10**^**−8**^	**6.97 × 10**^**− 8**^	**5.87 × 10**^**− 8**^	**7.80 × 10**^**−7**^	**2.07 × 10**^**− 8**^	**5.29 × 10**^**− 7**^

Significant associations are shown in bold face (*P* < 0.05)

*OR* odds ratio, *CI* confidence interval

*P-value of logistic regression analysis by adjusting for sex and age as covariates

**Previously identified CHB marker

### Cumulative genetic effects of CHB susceptible loci

In order to examine the detailed genetic effects of all 11 CHB susceptible loci including *rs12614* (*rs12614* of *CFB*; *rs9277535* of *HLA-DPB1*; *rs3077* of *HLA-DPA1*; *rs2856718* of *HLA-DQB1*; *rs7453920* of *HLA-DQB2*; *rs1419881* of *TCF19*; *rs1265163* of *OCT4*; *rs652888* and *rs35875104* of *EHMT2*; *rs9394021* and *rs2517459* of *VARS2-SFTA)* in a Korean population, an allele test was conducted for each SNP. The GRSs of the genotypes were calculated using the ORs from allele test (Table 3).

**Table 3:** Determination of Genetic Risk Score based on Allele test of CHB susceptible loci

Gene/Marker	Genotype distribution		Allele test			GRS^b^
	CHB (*n* = 955)	PC (*n* = 761)	Allele^a^	OR (95% CI)	*P*-value	
*CFB*/*rs12614*	CC(829)	CC(550)	C	1	–	1
	CT(91)	CT(142)	T	0.44 (0.33–0.57)	**6.46 × 10**^**− 10**^	0.44
	TT(3)	TT(7)				0.22
*HLA*-*DPB1*/*rs9277535*^c^	GG(420)	GG(184)	G	1	–	1
	GA(428)	GA(384)	A	0.49 (0.43–0.56)	**9.47 × 10**^**−24**^	0.49
	AA(107)	AA(193)				0.25
*HLA*-*DPA1*/*rs3077*^c^	GG(489)	GG(221)	G	1	–	1
	GA(382)	GA(385)	A	0.48 (0.41–0.55)	**1.84 × 10**^**−24**^	0.48
	AA(84)	AA(155)				0.24
*HLA*-*DQB1*/*rs2856718*^c^	CC(226)	CC(263)	C	1	–	1
	CT(418)	CT(373)	T	1.72 (1.50–1.97)	**3.10 × 10**^**−15**^	1.72
	TT(311)	TT(125)				3.44
*HLA*-*DQB2*/*rs7453920*^c^	GG(743)	GG(461)	G	1	–	1
	GA(196)	GA(275)	A	0.49 (0.41–0.60)	**1.07 × 10**^**−13**^	0.49
	AA(16)	AA(25)				0.25
*TCF19*/*rs1419881*^c^	TT(419)	TT(270)	T	1	–	1
	TC(424)	TC(361)	C	0.74 (0.64–0.85)	**3.46 × 10**^**−5**^	0.74
	CC(112)	CC(130)				0.37
*EHMT2*/*rs652888*^c^	TT(610)	TT(565)	T	1	–	1
	TC(300)	TC(188)	C	1.65 (1.37–1.99)	**5.14 × 10**^**−8**^	1.65
	CC(45)	CC(8)				3.3
*OCT4*/*rs1265163*^c^	GG(401)	GG(392)	G	1	–	1
	GC(439)	GC(299)	C	1.32 (1.14–1.53)	**1.36 × 10**^**−4**^	1.32
	CC(114)	CC(70)				2.64
*EHMT2*/*rs35875104*^c^	TT(878)	TT(650)	T	1	–	1
	TC(75)	TC(108)	C	0.53 (0.39–0.71)	**2.42 × 10**^**− 5**^	0.53
	CC(2)	CC(3)				0.27
*VARS2*-*SFTA*/*rs9394021*^c^	AA(303)	AA(189)	A	1	–	1
	AG(471)	AG(368)	G	0.73 (0.64–0.84)	**1.10 × 10**^**−5**^	0.73
	GG(177)	GG(203)				0.37
*VARS2*-*SFTA*/*rs2517459*^c^	GG(809)	GG(560)	G	1	–	1
	GA(139)	GA(193)	A	0.53 (0.43–0.67)	**3.71 × 10**^**−8**^	0.53
	AA(6)	AA(8)				0.27

Significant associations are shown in bold face (*P* < 0.05)

*CHB* chronic hepatitis B, *PC* population control, *OR* odds ratio, *CI* confidence interval, *GRS* genetic risk score

^a^Major and minor alleles were determined in all study subjects

^b^GRS was calculated by multiplying the number of minor alleles by effect size (OR) of the SNP

^c^Previously identified CHB marker in Korean population

**Fig. 1:**
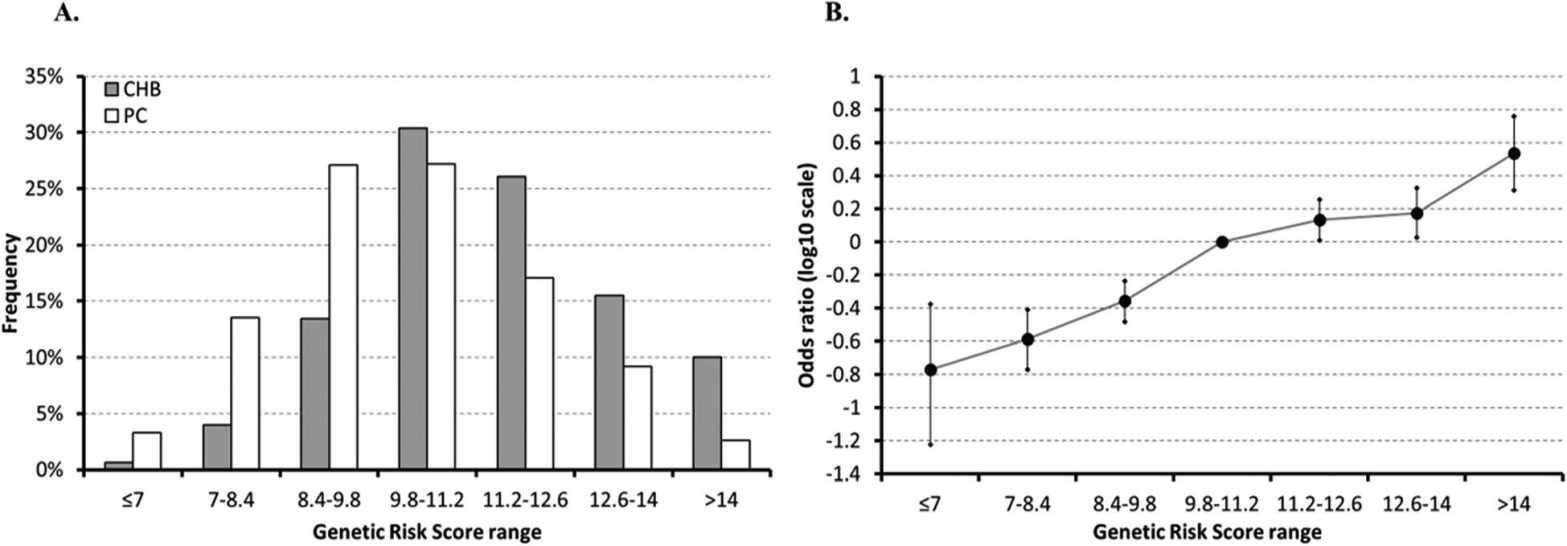
Combined genetic effects of eleven CHB genetic markers on the risk of CHB. **a** Comparison of GRS between CHB group and PC group. **b** Odds ratios of different GRS range in log10 scale. Median genetic risk score range (9.8–11.2) is used as a reference. CHB, chronic hepatitis B; PC, population control; GRS, genetic risk score

To elucidate the cumulative genetic effects of all 11 CHB loci in the study subjects, the cumulative GRSs were evaluated. The cumulative GRSs ranged from 5.24 (most protected group) to 17.38 (most susceptible group), and CHB patients showed significantly higher cumulative GRSs than did the healthy control subjects (Supplementary Table 4 and Fig. 1a). It was found that as cumulative GRSs increased, ORs significantly increased as well. In particular, individuals with GRSs of less than 7 showed an OR of 0.17 (log_10_ OR = − 0.77), while individuals with GRSs of over 14 showed an OR of 3.42 (log_10_ OR = 0.53) (Fig. 1b).

## Discussion

Hepatitis B, an infectious disease with a high rate of incidence in Asian populations [1], is a major cause of CHB, liver failure, liver cirrhosis, and HCC development, diseases which often result in death [21, 22]. Although the mechanisms underlying the different clinical results of HBV infection have not been fully understood, previous studies have linked a diverse range of factors such as viral strain, gender, age of infection, host immune system, and genetic information of the host, with risk of CHB [23]. When viral infection occurs, several immune-related genes are activated, leading to disease outbreak. According to a GWAS conducted on a Chinese population, a *CFB* genetic variant had significant association with risk of CHB [10]. In this study, we aimed to 1) validation of GWAS association signal SNP (*CFB rs12614*) on CHB susceptibility in a Korean population, and 2) identification of possible additional causal variants around GWAS association signal SNP on CHB susceptibility in a Korean population by fine-mapping of *CFB* region.

The complement system is composed of over 30 plasma proteins and is activated by microbes or antibodies which attached to microbes or other antigens [24, 25]. This system is an innate immune system that helps operate rapid responses against pathogenic invasions by opsonizing or recruiting inflammatory cells or pathogen lysis [26]. The complement activations occur through three pathways: the classical pathway, the lectin pathway and the alternative pathway. These pathways are worked through a cascade of enzymes reaction [25, 27]. CFB is essential to activate the complement system, particularly the alternative pathway that is against microbe invasion which includes viruses [28].

Previous Chinese studies have identified *CFB* genetic variants which have genetic effect on CHB risk. The most significant association was identified at *rs12614* of *CFB* (*P* = 1.28 × 10^− 34^ - 4.0 × 10^− 3^) [10, 11]. In this study, *rs12614* showed the same direction of genetic effect as found in previous Chinese studies. In order to validate the associations, we have conducted the validation analysis of *CFB* genetic variant, *rs12614*, using random sampling of the training and test sets from the subjects. As result, all training sets showed significant results and although not all test sets showed significant results due to small sample sizes in test sets, the trends of effects were the same directions (Supplementary Table 3). Moreover, *CFB rs12614* was significantly associated with risk of CHB in the HCC (−) CHB and the HCC (+) CHB groups (*P* = 6.60 × 10^− 8^ and 3.10 × 10^− 6^, respectively). However, there was no significant genetic effect on CHB-related HCC progression. Additionally, the *rs12614* C > T T allele was more frequently observed in the PC group than the CHB patients with a significance (OR = 0.43, *P*_corr_ = 2.36 × 10^− 8^). Considering that individuals with the non-synonymous variant (*rs12614* T allele) had significantly higher CFB expression than those with the *rs12614* C allele in the Chinese study, it can be seen that the *rs12614* may affect immune response by influencing the complement system when viral infection occurs [10].

The *rs12614* which is located on coding region of *CFB*, C to T allele change causes the amino acid change, arginine to tryptophan. To predict the effects of the *rs12614* amino acid change, we conducted in silico analysis using the PolyPhen-2 program (http://genetics.bwh.harvard.edu/pph2/index.shtml) [29]. The results demonstrated that this amino acid change is predicted to be probably damaging that means this substitution might be damaging with high confidence (Supplementary Figure 3A, [29]). Amino acid alignment from the program, arginine at position 32 is highly conserved among species (Supplementary Figure 3B). Additionally, protein structure prediction was performed using CFSSP: Chou & Fasman Secondary Structure Prediction Server (http://www.biogem.org/tool/chou-fasman/index.php) [30]. Changes in protein secondary structure of *rs12614* region, from coil structure to helix structure, by amino acid change from arginine to tryptophan were predicted. (Supplementary Figure 4). Protein function is closely related to the structure so that amino acids residue substitution can modify functional sites or protein interactions. And also disease-causing substitutions are more likely to occur at positions that is conserved throughout evolution [31], the *rs12614* C to T allele substitution may affect CFB functions. Because the alternative pathway is important to against pathogen invasion, an amino acid change in *CFB* important in the alternative pathway may affect the immune system to against hepatitis B virus invasion.

Some individuals are more susceptible to diseases while others are less susceptible. Identification of the genetic background is key to understand differences in individuals’ disease susceptibility, and that can potentially lead to the targeting of preventive measures at those who are at greatest risk [32]. The results of the conditional analysis conducted on the 10 previously identified markers indicated that *rs12614* can be used as a novel causal variant of CHB susceptibility. To elucidate its cumulative genetic effects, we used odd ratios of *rs12614* and previously identified 10 CHB markers. Consequently, CHB group showed higher GRSs than the PC group and the higher genetic risk scores range indicated higher odds ratios. These implies CHB patients are more likely to have higher scores than controls.

There is a sampling limitation in this study. While the ideal subjects for the control groups would be the people who are HBsAg (−) and anti-HBc (+) (spontaneously cleared), we used population controls with unknown responses to HBV infection. And some individuals in the control group still have a chance of progression to CHB when exposed to HBV. Although using the population controls in a case-control study may reduce statistical power, it is useful when it is difficult to obtain a sufficient number of disease controls.

## Conclusions

A non-synonymous variant, *rs12614* (Arg32Trp) of *CFB* was found to have significant associations with risk of CHB in a Korean population. Moreover, genetic effect of *rs12614* on CHB risk was independent of all known CHB risk loci, and *rs12614* can be used as possible causal variant of CHB susceptibility. Therefore, the results from this study may help in understanding and predicting genetic susceptibility to CHB in a Korean population.

## Supplementary Information

**Additional file 1: Supplementary Figure 1.** Gene map and LD plot of *CFB*. **A.** Gene map of *CFB* (*complement factor B*) on chromosome 6p21.33 (6 kb). Black blocks mean coding exons, and white blocks mean 5′ and 3′ UTRs. **B.** Linkage disequilibrium (LD) plot of *CFB* polymorphisms. Numbers in color boxes indicate |*D’*| values.

**Additional file 2: Supplementary Figure 2**. LD plot of *rs12614* and previously identified 10 CHB markers. LD plot of *CFB rs12614* and previously identified 10 CHB susceptibility markers. Numbers in black and white boxes indicate *r*^2^ values. LD structure was constructed by Haploview software.

**Additional file 3: Supplementary Figure 3.** In silico analysis of *CFB rs12614.* **A.** To predict amino acid change (Arg32Trp) in *CFB rs12614* affects protein function, HumDiv model analysis was conducted using PolyPhen-2 v2.2.2r398. Arg32Trp mutation is predicted to be probably damaging with a score of 0.985 (http://genetics.bwh.harvard.edu/pph2/index.shtml). **B.** Amino acids sequence alignment about diverse species of *CFB rs12614* was conducted using PolyPhen-2 v2.2.2r398 according to *UniProtKB/UniRef100* (http://genetics.bwh.harvard.edu/pph2/index.shtml). Shown are 75 amino acids surrounding the mutation position (marked with a black box).

**Additional file 4: Supplementary Figure 4.** Secondary structure prediction of CFB. To predict protein secondary structure when *CFB* allele change, CFSSP: Chou & Fasman Secondary Structure Prediction Server (http://www.biogem.org/tool/chou-fasman/index.php) was used. (A) Secondary structure of *C* allele (arginine) and (B) *T* allele (tryptophan) in *CFB rs12614*. H, E, T, and C indicate helix structure, sheet structure, turn and coil structure, respectively.

**Additional file 5: Supplementary Table 1.** Characteristics of study subjects.

**Additional file 6: Supplementary Table 2.** Genotype distribution of investigated CFB genetic polymorphisms among subjects investigated in this study.

**Additional file 7: Supplementary Table 3.** Association analysis of *rs12614* using the Training and Test sets.

**Additional file 8: Supplementary Table 4.** Combined genetic effects of eleven CHB susceptible loci.

## Data Availability

All statistic data generated or analyzed during this study are included in this published article and its supplementary information files.

## Abbreviations

CHB: Chronic hepatitis B
CFB: *Complement factor B*
SNP: Single nucleotide polymorphism
GRSs: Genetic risk scores
HBV: Hepatitis B virus
HCC: Hepatocellular carcinoma
GWASs: Genome-wide association studies
HLA: Human leukocyte antigen
*TCF19*: *Transcription factor 19*
*VARS2-SFTA2*: *Valyl-tRNA synthetase 2*-*surfactant associated 2*
*EHMT2*: *Euchromatin histone-lysine-methyltransferase 2*
PC: Population control
MAF: Minor allele frequency
LD: Linkage disequilibrium
HWE: Hardy-Weinberg Equilibrium
ORs: Odds ratios
