# Supplementary figures and images for: A non-synonymous variant rs12614 of complement factor B associated with risk of chronic hepatitis B in a Korean population
Source: BMC Med Genet. 2020 Dec 17;21:241. doi: 10.1186/s12881-020-01177-w (PMC7745368; doi:10.1186/s12881-020-01177-w)

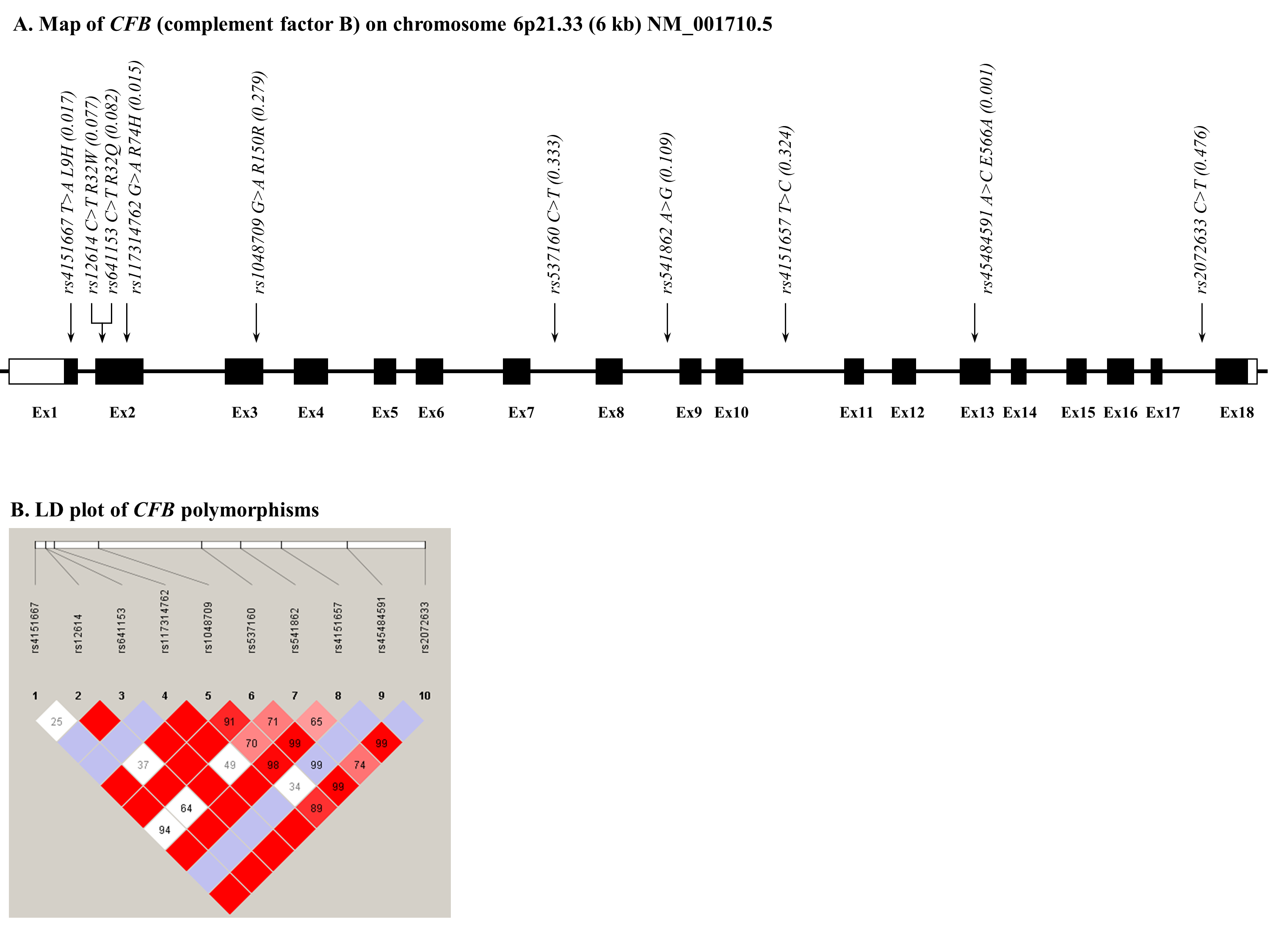

Supplement: Supplementary file 1 — Additional file 1: Supplementary Figure 1. Gene map and LD plot of CFB. A. Gene map of CFB (complement factor B) on chromosome 6p21.33 (6 kb). Black blocks mean coding exons, and white blocks mean 5′ and 3′ UTRs. B. Linkage disequilibrium (LD) plot of CFB polymorphisms. Numbers in color boxes indicate |D’| values. [file 12881_2020_1177_MOESM1_ESM.tif]

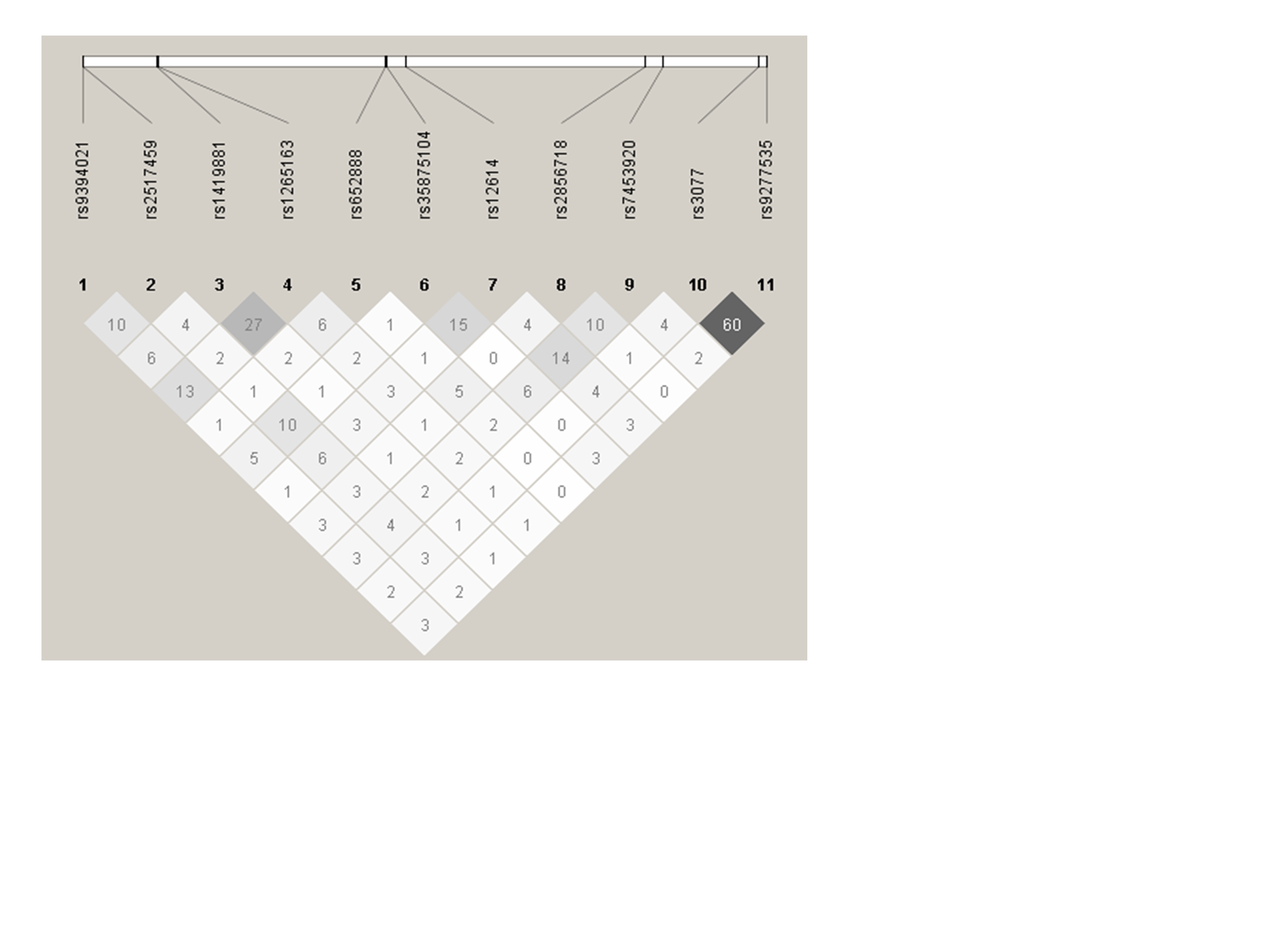

Supplement: Supplementary file 2 — Additional file 2: Supplementary Figure 2. LD plot of rs12614 and previously identified 10 CHB markers. LD plot of CFB rs12614 and previously identified 10 CHB susceptibility markers. Numbers in black and white boxes indicate r2 values. LD structure was constructed by Haploview software. [file 12881_2020_1177_MOESM2_ESM.tif]

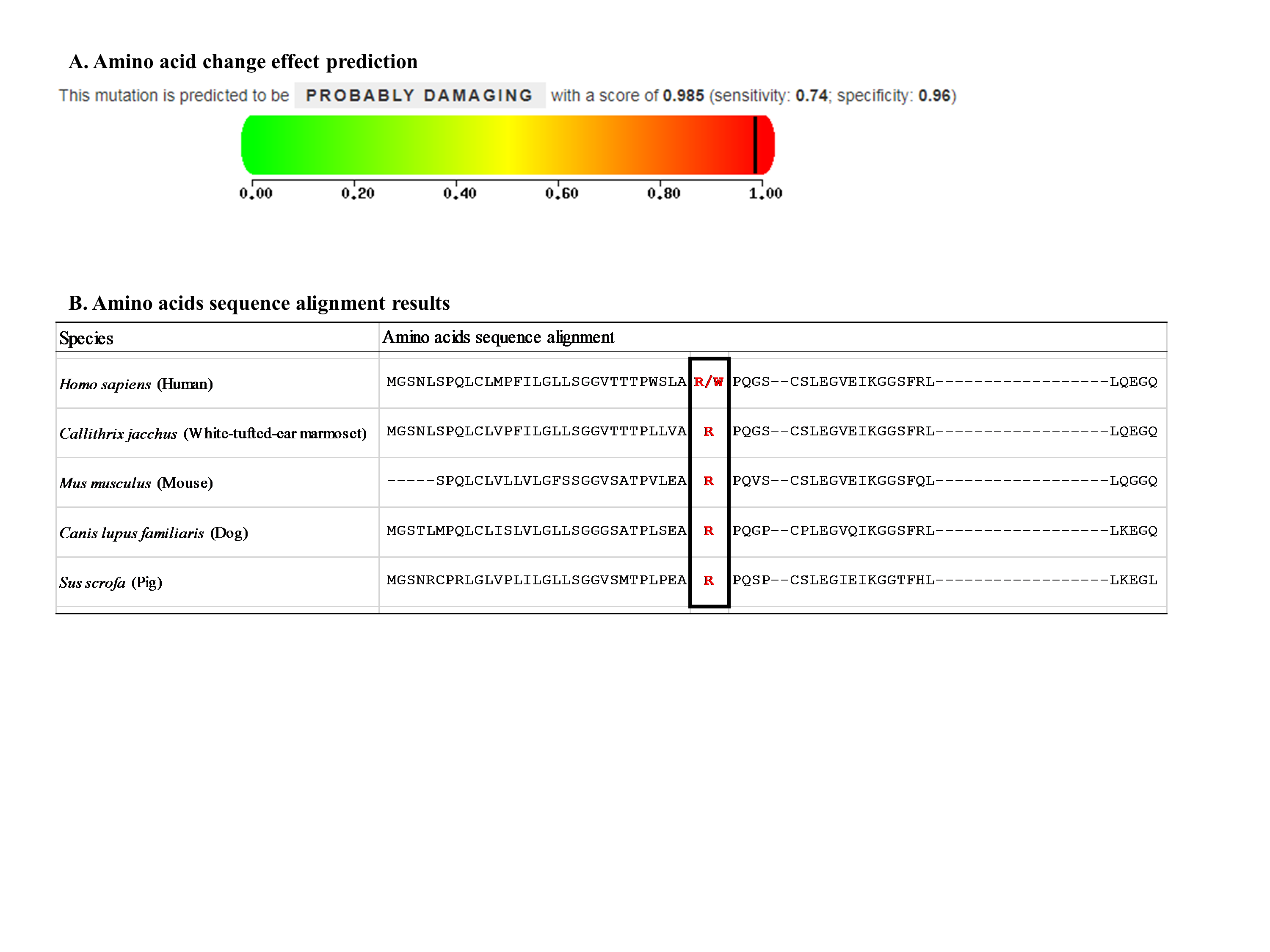

Supplement: Supplementary file 3 — Additional file 3: Supplementary Figure 3. In silico analysis of CFB rs12614. A. To predict amino acid change (Arg32Trp) in CFB rs12614 affects protein function, HumDiv model analysis was conducted using PolyPhen-2 v2.2.2r398. Arg32Trp mutation is predicted to be probably damaging with a score of 0.985 (http://genetics.bwh.harvard.edu/pph2/index.shtml). B. Amino acids sequence alignment about diverse species of CFB rs12614 was conducted using PolyPhen-2 v2.2.2r398 according to UniProtKB/UniRef100 (http://genetics.bwh.harvard.edu/pph2/index.shtml). Shown are 75 amino acids surrounding the mutation position (marked with a black box). [file 12881_2020_1177_MOESM3_ESM.tif]

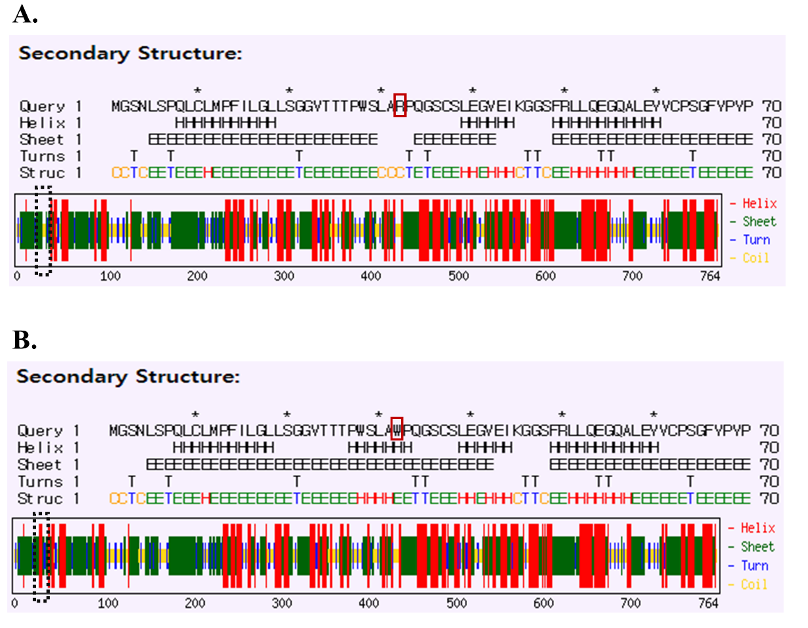

Supplement: Supplementary file 4 — Additional file 4: Supplementary Figure 4. Secondary structure prediction of CFB. To predict protein secondary structure when CFB allele change, CFSSP: Chou & Fasman Secondary Structure Prediction Server (http://www.biogem.org/tool/chou-fasman/index.php) was used. (A) Secondary structure of C allele (arginine) and (B) T allele (tryptophan) in CFB rs12614. H, E, T, and C indicate helix structure, sheet structure, turn and coil structure, respectively. [file 12881_2020_1177_MOESM4_ESM.png]
