# Supplementary material for: A non-synonymous variant rs12614 of complement factor B associated with risk of chronic hepatitis B in a Korean population
Source: BMC Med Genet. 2020 Dec 17;21:241. doi: 10.1186/s12881-020-01177-w (PMC7745368; doi:10.1186/s12881-020-01177-w)
Supplement: Supplementary file 5 — Additional file 5: Supplementary Table 1. Characteristics of study subjects. [file 12881_2020_1177_MOESM5_ESM.docx]

**Supplementary Table 1**. Characteristics of study subjects

| Characteristics | CHB | PC |
| --- | --- | --- |
| Number of samples | 955 | 761 |
| Gender ratio (Male:Female) | 1.97:1 | 1.17:1 |
| Age (mean ± SD) | 49.18 ± 11.40 | 52.23 ± 5.67 |
| HBsAg (positive rate) | 100% | - |
| HBsAb (positive rate) | 2.80% | - |
| Number of HCCs. | 296 | - |

PC, population control; CHB, chronic hepatitis B; SD, standard deviation; HCC, hepatocellular carcinoma
