# Supplementary material for: A non-synonymous variant rs12614 of complement factor B associated with risk of chronic hepatitis B in a Korean population
Source: BMC Med Genet. 2020 Dec 17;21:241. doi: 10.1186/s12881-020-01177-w (PMC7745368; doi:10.1186/s12881-020-01177-w)
Supplement: Supplementary file 6 — Additional file 6: Supplementary Table 2. Genotype distribution of investigated CFB genetic polymorphisms among subjects investigated in this study. [file 12881_2020_1177_MOESM6_ESM.docx]

**Supplementary Table 2**. Genotype distribution of investigated *CFB* genetic polymorphisms among subjects investigated in this study

| Marker | Chr:Position | Allele | Position (AA change) | Genotype | | | Heterozygosity | HWE | | |
| --- | --- | --- | --- | --- | --- | --- | --- | --- | --- | --- |
|  |  |  |  | C/C | C/R | R/R |  | Total | CHB | PC |
| *rs4151667* | 6:31946247 | T>A | Exon1 (Leu9His) | 1655 | 61 | 0 | 0.035 | 0.453 | 0.542 | 0.659 |
| *rs12614* | 6:31946402 | C>T | Exon2 (Arg32Trp) | 1379 | 233 | 10 | 0.144 | 0.963 | 0.765 | 0.516 |
| *rs641153* | 6:31946403 | C>T | Exon2 (Arg32Gln) | 1448 | 255 | 13 | 0.150 | 0.631 | 0.152 | 0.353 |
| *rs117314762* | 6:31946529 | G>A | Exon2 (Arg74His) | 1664 | 52 | 0 | 0.030 | 0.524 | 0.694 | 0.605 |
| *rs1048709* | 6:31947158 | G>A | Exon3 (Arg150Arg) | 892 | 689 | 135 | 0.403 | 0.903 | 0.728 | 0.814 |
| *rs537160* | 6:31948623 | C>T | Intron7 | 748 | 792 | 176 | 0.444 | 0.111 | 0.225 | 0.295 |
| *rs541862* | 6:31949174 | A>G | Intron8 | 1448 | 255 | 13 | 0.150 | 0.631 | 0.152 | 0.353 |
| *rs4151657* | 6:31949763 | T>C | Intron10 | 786 | 753 | 177 | 0.437 | 0.866 | 0.872 | 0.615 |
| *rs45484591* | 6:31950691 | A>C | Exon13 (Glu566Ala) | 1712 | 4 | 0 | 0.002 | 0.961 | 0.974 | 0.971 |
| *rs2072633* | 6:31951801 | C>T | Intron17 | 462 | 872 | 382 | 0.499 | 0.443 | 0.419 | 0.786 |

C/C, C/R, and R/R refer to the common homozygote, heterozygote, and minor homozygote, respectively.

Chr; chromosome, AA, amino acid; HWE, Hardy-Weinberg equilibrium *P*-value; PC, population control; CHB, chronic hepatitis B
