# Supplementary material for: A non-synonymous variant rs12614 of complement factor B associated with risk of chronic hepatitis B in a Korean population
Source: BMC Med Genet. 2020 Dec 17;21:241. doi: 10.1186/s12881-020-01177-w (PMC7745368; doi:10.1186/s12881-020-01177-w)
Supplement: Supplementary file 7 — Additional file 7: Supplementary Table 3. Association analysis of rs12614 using the Training and Test sets. [file 12881_2020_1177_MOESM7_ESM.docx]

**Supplementary Table 3. Association analysis of *rs12614* using the Training and Test sets**

| Group | | Number of Subjects | | |  | *rs12614* |  |
| --- | --- | --- | --- | --- | --- | --- | --- |
|  |  | Case | Control | Total |  | OR (95% CI) | P* |
| Training Set | Set 1 | 858 | 686 | 1544 |  | 0.45 (0.33-0.61) | **2.46E-07** |
|  | Set 2 | 859 | 685 | 1544 |  | 0.46 (0.34-0.61) | **2.29E-07** |
|  | Set 3 | 852 | 692 | 1544 |  | 0.43 (0.32-0.59) | **5.20E-08** |
|  | Set 4 | 860 | 684 | 1544 |  | 0.41 (0.30-0.56) | **1.00E-08** |
|  | Set 5 | 850 | 694 | 1544 |  | 0.38 (0.28-0.52) | **6.78E-10** |
|  | Set 6 | 861 | 683 | 1544 |  | 0.46 (0.34-0.62) | **4.31E-07** |
|  | Set 7 | 854 | 690 | 1544 |  | 0.44 (0.32-0.59) | **7.21E-08** |
|  | Set 8 | 851 | 693 | 1544 |  | 0.44 (0.33-0.59) | **8.54E-08** |
|  | Set 9 | 862 | 682 | 1544 |  | 0.42 (0.31-0.57) | **1.77E-08** |
|  | Set 10 | 857 | 687 | 1544 |  | 0.46 (0.34-0.63) | **5.12E-07** |
|  |  |  |  |  |  |  |  |
| Test Set | Set 1 | 97 | 75 | 172 |  | 0.33 (0.13-0.79) | **0.02** |
|  | Set 2 | 96 | 76 | 172 |  | 0.21 (0.05-0.70) | **0.01** |
|  | Set 3 | 103 | 69 | 172 |  | 0.82 (0.28-2.57) | 0.72 |
|  | Set 4 | 95 | 77 | 172 |  | 0.72 (0.29-1.79) | 0.47 |
|  | Set 5 | 105 | 67 | 172 |  | 2.44 (0.75-11.0) | 0.18 |
|  | Set 6 | 94 | 78 | 172 |  | 0.31 (0.12-0.73) | **0.009** |
|  | Set 7 | 101 | 71 | 172 |  | 0.42 (0.15-1.14) | 0.09 |
|  | Set 8 | 104 | 68 | 172 |  | 0.40 (0.14-1.13) | 0.08 |
|  | Set 9 | 93 | 79 | 172 |  | 0.64 (0.23-1.84) | 0.40 |
|  | Set 10 | 98 | 74 | 172 |  | 0.22 (0.07-0.61) | **0.006** |

A total of 10 sets were determined for validation analysis.

For each set, study subjects were divided at a ratio of 9 (training set) to 1 (test set).

**P*-value of logistic analysis under additive model by adjusting for sex and age as covariates.

OR, odds ratio; CI, confidence interval.
