# Supplementary material for: A non-synonymous variant rs12614 of complement factor B associated with risk of chronic hepatitis B in a Korean population
Source: BMC Med Genet. 2020 Dec 17;21:241. doi: 10.1186/s12881-020-01177-w (PMC7745368; doi:10.1186/s12881-020-01177-w)
Supplement: Supplementary file 8 — Additional file 8: Supplementary Table 4. Combined genetic effects of eleven CHB susceptible loci. [file 12881_2020_1177_MOESM8_ESM.docx]

**Supplementary Table 4**. Combined genetic effects of eleven CHB susceptible loci

| Risk score range | Number of subjects (%) | | OR(95%CI) | *P*-value* |
| --- | --- | --- | --- | --- |
|  | CHB | PC |  |  |
| ≤ 7 | 6 (0.63%) | 25 (3.29%) | 0.17 (0.06-0.42) | **1.00×10^-4^** |
| 7 - 8.4 | 38 (3.98%) | 103 (13.53%) | 0.26 (0.17-0.39) | **2.31×10^-10^** |
| 8.4 - 9.8 | 128 (13.40%) | 206 (27.07%) | 0.44 (0.33-0.58) | **1.94×10^-8^** |
| 9.8 - 11.2 | 290 (30.37%) | 207 (27.20%) | 1 | - |
| 11.2 - 12.6 | 249 (26.07%) | 130 (17.08%) | 1.36 (1.03-1.80) | **2.70×10^-2^** |
| 12.6 - 14 | 148 (15.50%) | 70 (9.20%) | 1.50 (1.07-2.11) | **1.62×10^-2^** |
| > 14 | 96 (10.05%) | 20 (2.63%) | 3.42 (2.05-5.72) | **2.62×10^-6^** |

**P*-value of logistic regression analysis by adjusting for sex and age as covariates.

Median risk score range (9.8 – 11.2) is used as a reference.

Significant associations are shown in bold face.

CHB, chronic hepatitis B; PC, population control; OR, odds ratio; CI, confidence interval.
